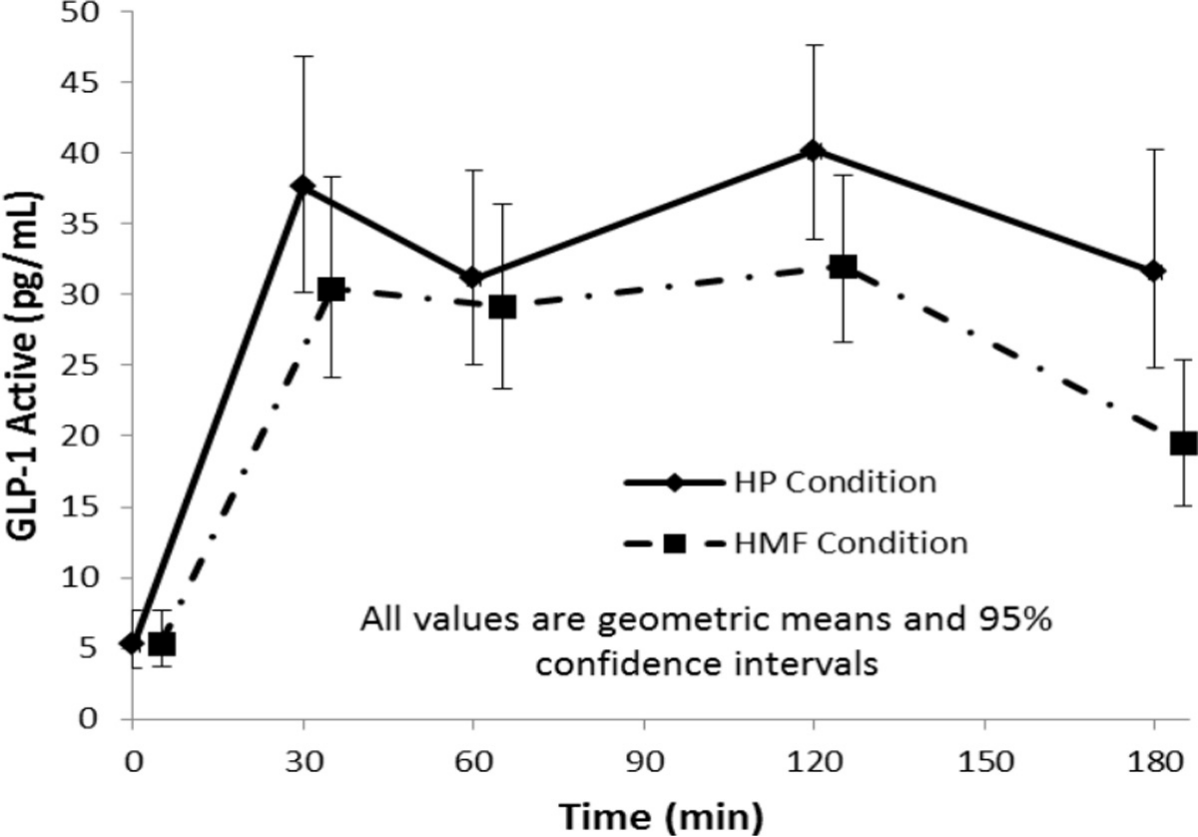# The effect of meal composition on postprandial glucagon-like peptide-1 response in overweight/obese participants

**DOI:** 10.1186/1550-2783-12-S1-P12

**Published:** 2015-09-21

**Authors:** Brian Franklin, Beverley Adams-Huet, Melody Phillips, Joel Mitchell, Brooke Bouza, Manall Jaffery, Alex Villanueva, Shane Jenke, Justin Repshas, Leighsa Brace, Henry Aleck, Aaron Caldwell, Elizabeth Sanders, Lyn Dart, Meena Shah

**Affiliations:** 1 Department of Kinesiology, TCU, Fort Worth, TX 76129, USA; 2 Department of Clinical Sciences, UT Southwestern Medical Center at Dallas, Dallas, TX 75390, USA; 3 Department of Nutritional Sciences, TCU, Fort Worth, TX 76129, USA

## Background

Glucagon-like peptide-1 (GLP-1) is an incretin hormone secreted in the intestine in response to food intake. GLP-1 may be responsible for nearly 50% of insulin secretion. Postprandial GLP-1 secretion may be impaired in overweight/obese (OW/O) individuals and in patients with type-2 diabetes (T2D). Meals high in protein (HP) or high in monounsaturated fat (HMF) may increase GLP-1 response. However, there are no studies directly comparing HP with HMF meals on postprandial GLP-1 response.

## Methods

Twenty-four OW/O participants (male/female: 12/12; age: 38.7 ± 15.3 (mean ± standard deviation) years; BMI: 31.6 ± 4.0kg/m^2^) were studied. Participants consumed a HMF and a HP meal in a random order at least 4 days apart. The HMF meal contained 35.2% energy from fat and 20.7% from monounsaturated fat and the HP meal contained 31.9% energy from protein. Energy and carbohydrate content were similar across meals. Blood samples were collected in the fasting and postprandial (30, 60, 120, and 180 min) states and analyzed for GLP-1 (active and total), insulin, glucagon, C-peptide, and glucose. A mixed effects repeated measures analysis model was used to examine the effect of meal composition on the outcome variables.

## Results

There were statistically significant (p < 0.01) time and time by meal composition interaction effects on active GLP-1 (see figure). Also found were statistically significant (p < 0.01) time, meal composition, and time by meal composition interaction effects on total GLP-1, insulin, C-peptide, and glucagon. The responses were higher on the HP compared to the HMF meal (p < 0.05) for active and total GLP-1 and C-peptide at 120 and 180 min, insulin at 60, 120, and 180 min, and glucagon at 30, 60, 120, and 180 min. There was a significant time (p < 0.0001) but not meal composition (p = 0.14) or time by meal composition interaction (p = 0.83) effect on blood glucose.

## Conclusions

Postprandial GLP-1, insulin, C-peptide, and glucagon responses were higher on the HP compared to the HMF meal but there was no difference in blood glucose response by meal composition. Future studies comparing meal composition on GLP-1 need to be longer in duration and in participants with T2D.

**Figure 1:**